# Effects of Vision Therapy on Bilateral Amblyopia Unresponsive to Conventional Treatment: A Retrospective Comparative Study

**DOI:** 10.3390/children9020205

**Published:** 2022-02-05

**Authors:** Yu-Te Huang, Hui-Ju Lin, Wen-Ling Liao, Yi-Yu Tsai, Yi-Ching Hsieh

**Affiliations:** 1 Department of Ophthalmology, China Medical University Hospital, China Medical University, Taichung 40447, Taiwan; tonyhuang791112@gmail.com (Y.-T.H.); d2396@mail.cmuh.org.tw (H.-J.L.); yiyutsai@seed.net.tw (Y.-Y.T.); 2 Center for Personalized Medicine, China Medical University Hospital, Taichung 40447, Taiwan; T12678@mail.cmuh.org.tw; 3 Graduate Institute of Integrated Medicine, China Medical University, Taichung 40447, Taiwan

**Keywords:** amblyopia, dichoptic training, orthoptic therapy, perceptual learning, vision therapy

## Abstract

Background: In this study we aim to determines the effect of our vision therapy program for 7- to 10-year-old patients who exhibit bilateral amblyopia that is no longer responsive to conventional treatment. Methods: Children with bilateral amblyopia between the ages of 7 and 10 treated with vision therapy at the China Medical University Hospital between 2016 and 2019 were retrospectively reviewed. Age and visual acuity-matched bilateral amblyopes are included as a control group. The visual acuity for both groups showed no improvement for more than 3 months with part-time patching and full refraction correction. The initial and final visual acuity, stereopsis, and refractive status were analyzed. Results: Here, 15 cases were included as the treatment group and 16 cases as a control group. At the endpoint, the study group shows a significant improvement in BCVA, with a mean of 0.32 ± 0.15 logMAR (3 lines improvement) versus 0.003 ± 0.19 logMAR (nearly no improvement) for the control group (*p* < 0.001). The benefits of treatment are most obvious in the first 3 months after treatment (*p* < 0.001) and last until the end point. Stereoacuity also improves from 190.00 ± 163.34 to 85.00 ± 61.24 arc seconds, which is a 55.26% improvement. Conclusions: Vision therapy, comprising orthoptic therapy, perceptual learning and dichoptic training, is a successful program for increasing visual acuity and stereoacuity in 7- to 10-year-old children with bilateral amblyopia that is unresponsive to conventional treatment.

## 1. Introduction

Amblyopia is a form of neurodevelopmental disorder of the visual cortex and the visual pathway. The definition varies but it is generally defined as decreased visual acuity in the absence of any explicable pathological or structural ocular defects [1]. The condition is usually unilateral and occurs in 0.73 to 1.9% of pre-school children and 1.0 to 5.5% of older school children, depending on the definition and the population [2,3,4]. The prevalence of bilateral amblyopia varies considerably and various studies shows that it accounts for 5% to 41% of all amblyopia [3,4,5,6].

Bilateral amblyopia is considered to be resulting from deprivation in the cerebral cortex due to the input of blurred retinal images. Few studies focus on bilateral amblyopia and feature a small number of cases and a relatively short follow-up period [7,8]. In contrast with monocular amblyopia, the prognosis without treatment is worse and age or learning have little effect. In a prospective study conducted by the Pediatric Eye Disease Investigator Group (PEDIG), after one year of conventional treatment (optical and patching treatment), 13% of children still suffered from a monocular acuity ≤ 20/40 in both eyes and 34% of children had a monocular acuity of ≤20/40 in one eye [8].

In the past decades, the concept of that amblyopia is a binocular disorder that involves interocular suppression, leads to the deeper research on binocular dysfunctions, such as impaired stereoacuity, motion perception and spatial localization [9]. In support of this evidence, new vision therapy with increasingly binocular approaches has been developed. The strategy is rebalancing the strength of the visual input for the two eyes to overcome interocular suppression and encourage simultaneous perception. These methods include orthoptic therapy, perceptual learning and dichoptic training.

In this present study, we mainly focus on children with bilateral refractive amblyopia who are aged from 7 to 10 years old and unresponsive to conventional treatments (optical and patching treatment). The aim of this study is to determine the effect of our vision therapy program (a combination of orthoptic, perceptual and dichoptic training) on visual acuity and stereo vision in these amblyopic children.

## 2. Materials and Methods

### 2.1. Ethical Approval

This retrospective, interventional, comparative case series study was conducted at China Medical University Hospital (CMUH) between 1 April 2016 and 30 November 2019. The study was performed in accordance with the World Medical Association’s Declaration of Helsinki and the study design was approved by the Institutional Review Board of CMUH (IRB number: CMUH110-REC2-002). Owing to the retrospective design of the study and the use of deidentified patient information, the review board waived the need for written informed consent.

### 2.2. Study Population

Medical records of children of 7 to 10 years old who were diagnosed as having amblyopia due to anisometropia, or isometropia were reviewed. The clinical practice protocol in our hospital was to offer visual training programs to patients who had been no visual acuity improvement in the past 3 months under conventional therapy (full refractive correction with training at home or alterative patching for 4 h). Other inclusion criteria were bilateral amblyopia, with a monocular best-corrected-visual acuity (BCVA) of 0.2 logMAR (20/32) to 1.0 logMAR (20/200) in both eyes, and follow-up for ≧6 months after beginning vision therapy. A group of age and BCVA-matched control group were selected. The control group followed the same treatment protocol except they refused to join the additional vision therapy program.

The exclusion criteria are lost follow up, missed more than one session of vision therapy, strabismus or other coexisting ocular disease, previous ocular surgery, previous vision therapy, premature birth of more than 8 weeks and developmental delay.

### 2.3. Clinical Practice Protocol

All children treated for amblyopia in our hospital followed the same protocol. They wore full optical correction. Additional patching was given for 4 h per day in patients who have an interocular difference (IOD) of 2 lines (0.18 logMAR) or more. They all performed additional amblyopia training exercises at home for 30 min per day, such as a maze and connect the dots. The only different was vision therapy were given to the study group in the office for 1 h once a week for at least 3 months.

Distance BCVA and cycloplegic refraction were measured at baseline and at the 3-month, 6-month, and 9-month visits and 3 months after cessation of treatment. Refractive error was measured using an autorefractor Topcon RM-8900 before and after administration of cycloplegics. BCVA was determined using the Landolt C chart at 5 m monocular. Luminance values were 160 to 200 cd m^−2,^ as recommended for standard measurements. Stereoacuity was measured using the Stereo Butterfly Test (Stereo Optical Co., Inc., Chicago, IL, USA) according to the manufacturer’s instructions. At the baseline visit, a full eye examination was conducted by the same pediatric ophthalmologist (Y.C.H.) with anterior segment examination, ocular motility, a cover-uncover test and fundus examination.

### 2.4. Training Apparatus

Most of the training apparatus was produced by the Bernell Corporation (Mishawaka, IN, USA)) [10]. Orthoptic training used antisuppression charts, red-green bar reader, single and double Aperture Rule, Keystone Eccentric Circles, Lifesaver cards, Corian cheiroscope, Wolff standup cheiroscope, vectogram, mirror stereopscope, single oblique stereoscope, Keystone correct-eye scope, pencil push-ups, accommodative flippers, Brock string and Hart chart. These tools increase vergence activity, accommodative activity, antisuppression training and simultaneous vision. Perceptual learning includes manual rotator, Marsden ball, Wayne saccadic fixator, Space fixator, and Eyeport vision training system. These tools are used for perceptual training, peripheral vision awareness and eye–hand coordination exercises. In dichoptic training, combined with red-green goggles and tranaglyphs are the most widely used tools. The detailed explanation of each instrument can be found via the website or the operating manual of the device [10]. For example, Cheiroscope is a device to enhance binocular stability and anti-suppression. The teacher will encourage the children to look into the cheiroscope, trace the target, and use a pencil to draw the target simultaneously. If the children stated some parts of the picture is missing, we can assume suppression is present. On the other hand, if the teacher finds out the children shift their hands while attempting to trace, or actual drifts of drawing pictures, we can suppose binocularity is existing. As for aperture rule, the instruments contain 12 cards with varying disparities. Each cards include a combination of second-degree (flat fusion) and third-degree target (stereopsis). Base on the children’s response, we can monitor and record his/her binocularity. If the children report that they see two targets or double vision, the binocular vision is not adequate enough. We can also differentiate suppression or intact binocularity based on the answers the children give us.

### 2.5. Training Programs

Each one-hour training program was divided into three parts: orthoptic therapy, then dichoptic training, and finally perceptual learning. Not all the participants used the same apparatus but the apparatus is from the same category. There are general principles in treatment program to maintain flexibility instead of hard and fast rules. This design has several considerations. First, the acceptability is different among instruments were chosen in terms of their suitability to the child. For example, in orthoptic therapy, Keystone Eccentric Circles, Lifesaver cards, and Corian cheiroscope are the easiest. So, these three instruments are always given first. When the patients can perform well of these three instruments or lose interest, second line apparatus such as vectogram, mirror stereopscope, and single oblique stereoscope are prescribed. Then, the third line instruments such as pencil push-ups, accommodative flippers and Brock string are slightly more difficult to accomplish. Aperture Rule, antisuppression charts and Hart chart are considered the most complicated and required better concentration and fusion ability which will be given mostly in the last month of training. Second, perceptual learning uses tools that are more interesting for children so this comprised the final part of the one-hour program. In perceptual learning, manual rotator mostly comes first, followed by Marsden ball, Wayne saccadic fixator and Space fixator. Eyeport vision training system which require higher vergence, accommodative facility and reading performance, is considered to be prescribed later according to the performance. Last, using different types of apparatus in a single category allowed the children to engage and encouraged compliance with the training regimen.

### 2.6. Statistical Analysis

All statistical data was analyzed using SPSS Statistics 24 (IBM Corporation, Somers, NY, USA). Continuous data is presented as a mean and standard deviation and categorical data is presented as a proportion. The t-test was used to compare the mean values of continuous variables and a Chi-squared test was used to compare the frequency of categorical variables between the two groups. Mann-Whitney U test and chi-square tests were, respectively, used to determine the difference in continuous variables and categorical variables. Wilcoxon signed rank test was used to compare the difference in VA between the baseline and the follow-up for each group. A *p*-value < 0.05 represents a statistically significant difference.

## 3. Results

### 3.1. Demographic and Clinical Characteristics of Patients

In this case, 15 amblyopic children aged 7–10 years (mean 7.60 ± 0.74; 9 females) were included in the study group. The control group included 16 amblyopia children aged 7–10 years (mean 7.63 ± 0.72; 5 females). Baseline characteristics are listed in Table 1. There were 11 isometropic children in the case group and the control group. There were 3 patients in the control group and 2 patients in the study group received additional patching 4 h a day. In the study group, mild amblyopia (range, <0.3 logMAR) was present in 6 children (40.0%), moderate amblyopia (range, 0.3–0.7 logMAR) was present in 8 (53.3%) and severe amblyopia (range, >0.7 logMAR) was present in 1 (6.7%). In the control group, mild amblyopia was present in 3 children (18.7%), moderate amblyopia (range, 0.3–0.7 logMAR) was present in 10 (62.5%) and severe amblyopia (range, >0.7 logMAR) was present in 3 (18.7%). About the refractive status, most of the cases in control group are myopic (93.3%) and 66.7 percent of the cases are highly myopic (<−5 D). In addition, the mean value of total follow-up period in study group is 6.4 months, and the control groups was 6.31 months (*p* = 0.95).

### 3.2. Visual Acuity

**Figure 1:**
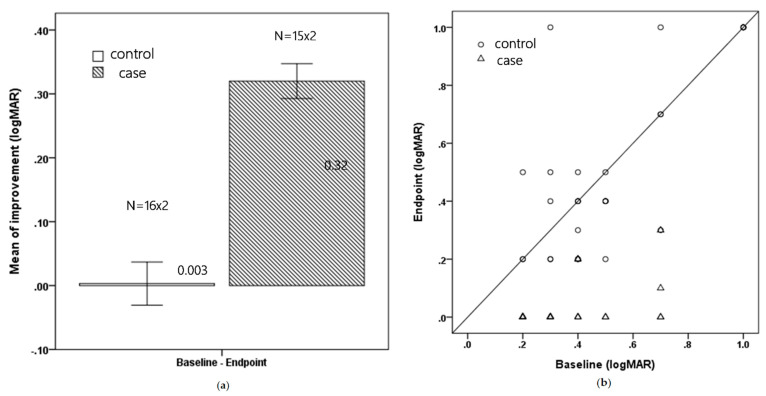
(**a**) Mean improvement in BCVA for the control and treatment groups (logMAR) (Mean ± Standard error) (**b**) The total change in BCVA for each patient (logMAR).

At the endpoint visit, subjects in the study group showed a significantly greater improvement in BCVA, with a mean of 0.32 ± 0.15 logMAR (3 lines improvement) versus 0.003 ± 0.19 logMAR (nearly no improvement) (*p* < 0.001). The average treatment duration was 5.7 months (range, 3–12 months) for the case group and 6.19 months (range 3–12 months) for the control group. Figure 1a shows the mean improvement for both groups and Figure 1b shows the change in each patient.

### 3.3. Timing of Visual Improvement

In the first 3 months of treatment, the mean improvement in BCVA was 0.28 ± 0.14 log MAR for the case group (*n* = 30 eyes) and 0.03 ± 0.12 logMAR for the control group (*n* = 24 eyes) (*p* < 0.001). At the 6th month visit, the mean improvement in BCVA was 0.27 ± 0.15 log MAR (*n* = 12 eyes) and −0.03 ± 0.22 logMAR (*n* = 16 eyes) (*p* < 0.001). At the 9th month, the improvement in BCVA was 0.39 ± 0.20 log MAR (*n* = 8 eyes) and 0.05 logMAR (*n* = 8 eyes) (*p* = 0.001). At the 12th month, the improvement in BCVA was 0.45 ± 0.13 log MAR (*n* = 4 eyes) and −0.03 ± 0.14 logMAR (*n* = 8 eyes) (*p* < 0.001). Of 22 eyes, 68.75% in the control group showed no improvement, whereas every eye in the study group showed better visual acuity. Table 2 shows the improvement in logMAR acuity from the baseline to the endpoint visit for both groups.

Table 3 shows the difference in BCVA between the baseline and follow-up visits for both groups. At the endpoint visit, the study group shows a significant mean gain of logMAR acuity (*p* < 0.001) but there is no significant difference in the control group (*p* = 0.613). For each 3-month follow-up visit, the study group shows a significant improvement in visual acuity, but not the control group.

### 3.4. Stereoacuity

**Figure 2:**
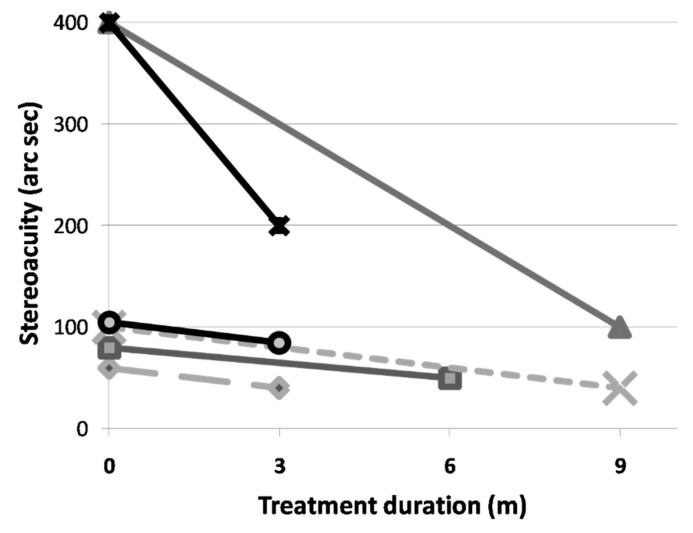
The individual improvement in stereoacuity after vision therapy.

Only 6 cases in the study group had complete and reliable stereoacuity data. All 6 cases showed an improvement in stereoacuity. The average stereoacuity improves from 190.00 ± 163.34 to 85.00 ± 61.24 arc seconds. This represents a 55.26% improvement. Due to the small number of data, the results are not statistically significant (Figure 2). Due to the retrospective nature of our study, only two patients in the control group could acquire completed stereoacuity data. One patient showed stereoacuity improvement from 400 arc seconds to 200 arc seconds during 9-month follow up period. Another patient with severe amblyopia (initial VA:20/200 in both eyes) demonstrated with no stereoacuity improvement (still 200 arc seconds) during 12-month follow up period.

Subjects in the study group suffered no adverse events, such as new tropia or diplopia, during the treatment period. There was no decline or further improvement in vision 3 months after the cessation of treatment.

## 4. Discussion

To the best of our knowledge, this is the first amblyopia study to focus primarily on the effect of vision therapy in children with bilateral amblyopia that is unresponsive to conventional treatment. This is a relatively rare subgroup for this condition because optical correction is a very effective treatment strategy for bilateral amblyopia.

A multicenter retrospective study by Klimek et al. reported that for 36 cases with bilateral refractive amblyopia, 83% of the eyes had final visual acuity of 0.5 (20/40) or better and 48% of children eventually achieved a visual acuity of 0.8 (20/25) after a mean follow-up of 3.3 years. (Mean age of 5.1 years old) [8]. Another study by Ziylan et.al reported that 31 children achieved a BCVA of 20/40 or better in 83.9% and 48.8% had a BCVA better than 20/25 after a mean follow-up of 55.8 months. (Mean age of 5.5 years old) [8]. Another study of 28 cases from Asia reported a mean VA at baseline of 0.39 logMAR, which significantly improved to 0.21 logMAR at one month and 0.02 logMAR at one year under optical treatment [11]. 

Given the paucity of literature on bilateral amblyopia and the relatively good prognosis, no previous studies focus on the management of an unresponsive group. Our study shows that this vision therapy gives excellent results. The mean improvement in VA is 0.32 logMAR (3 lines improvement) for the treatment group and 0.003 logMAR (almost no improvement) for the control group. This result confirms the unresponsive nature of the control group. In addition, we only include patients of 7 to 10 years of age who are previously believed optical treatment are less effective for this age group.

The explanation of unresponsiveness to conventional treatment is unclear but age and the refractive status play a significant role. Most studies that achieve better results were composed of high proportion of hyperopic patients. For our study group, the refractive status is mostly myopic (93.3%) and 66.7 percent of the cases are highly myopic (<−5 D). This condition might hinder the efficacy of optical treatment. Similar results were reported by previous studies, which noted that a large refractive error is a were risk factor for a poor prognosis for conventional treatment [7,12]. Further study is required but we propose that bilateral amblyopia, especially when accompanied with myopia, is more effectively treated using vision therapy than conventional treatment if the initial response to conventional treatment is suboptimal. 

Vision therapy is defined as an attempt to improve visual skills and abilities by optometrists initially. There are 2 main categories, including orthoptic vision therapy (Some studies further divided dichoptic training from this category) and behavior/perceptual vision therapy to improve binocular visual function. Two review articles support the use of vision therapy for treatment of amblyopia [13,14]. Hernández-Rodríguez demonstrated that vision therapy is a promising option for the treatment of children and teenagers with anisometropic amblyopia [13]. In adult amblyopia, perceptual vision therapy can enhance VA and visual performance [15,16]. 

Each component (orthoptic, perceptual and dichoptic training) of this combination therapy increases visual performance. Orthoptic therapy, despite lacking of strong evidence to exert alone in amblyopic treatment, it has been suggested as an adjuvant therapy that accelerates recovery, decreases equivalent input noise and increases efficiency [17,18]. In addition, anti-suppression, vergence and accommodative activity and simultaneous vision are all considered important for various visual tasks for children. Perceptual learning uses intensive and sustained practice or stimulus to increase the plasticity of a normal visual system [19,20]. It restores monocular visual acuity and more importantly, long standing stereopsis and interocular balance [9,17,18,21]. Some studies show that a reduction of lateral inhibition in the early stages of visual processing, reduction of internal noise that is inherent to amblyopia or a higher-level rule-based cognitive learning process is the underlying mechanism [22]. 

Considering amblyopia involves abnormal binocular interaction and interocular suppression, dichoptic training was developed and thought to be a relatively novel concept. The improvement in visual acuity varies for different studies and the instruments and compliance are the most important factors for dichoptic training [17,19,22,23,24]. Since it trains dichotically, the strength would be improving stereoacuity. The study by Liu et.al quantified with a more of 27% stereoacuity beyond the 55% gain from previous monocular training [24]. However, most subjects in previous studies exhibit unilateral amblyopia so the results are not applicable to our case series. Further studies are required to stratify the effect of vision therapy on bilateral amblyopia.

In our preliminary study shows that the vision training program also has significant effects in terms of unilateral amblyopia children. However, the total net visual gain is greater for bilateral cases than for unilateral cases (3 lines versus 2 lines). These results are as expected because the vision therapy for this study involves binocular training. This concept could be further strengthened by the improvement in stereopsis. Due to the retrospective nature of this study, stereoacuity in our studies were not completed. However, there is an obvious improvement (190.00 to 85.00 arc seconds) and these results were also better than those for unilateral cohorts. The total improvement is 55.26%, which is similar to the results for others studies that use dichotic training, which are around 64.2% to 68.2% [23,24].

This training program also accelerates recovery. Shoshany et al. treated 98 bilateral amblyopia cases using optical correction and occlusion and noted the greatest improvement occurred within 12- to 18-months [25]. These results are similar to our experience in the era of conventional treatment. For this study, visual acuity improves significantly in the first 3 month and improvement is sustained over the entire follow-up period.

Another strength of our vision therapy program is that we can assure the compliance. The compliance of occlusion is hard to define and usually low due to a range of factors including lengthy treatment periods, forced to use the eye with poor vision, cosmetic issue, skin discomforts. Previous studies showed the compliance of occlusion were low, ranging from 43 to 57% [26,27]. In addition, the good results of our program reconfirm the importance of compliance. 

Our control group is set for age and BCVA-matched, so the depth of amblyopia and age are not significantly different between two groups (*p* = 0.924 and 0.3342, respectively). However, the study group composes of more myopic patients, especially high myopic patients (66.7%). Due to scarcity of bilateral amblyopia patients, we cannot include proper number of refractive status-matched patients into our control groups. However, we still think our study is valid because previous studies have confirmed myopic amblyopic children had the worse prognosis, followed by hyperopic, and emmetropic patients [28,29]. We believed our training program will be more effective if refractive status is matched between two groups. 

The comparisons of characteristics before conventional treatment of amblyopia between two groups was not completed due to the retrospective nature of the study. There are 5 children in the study group (33%) and 7 children (43.8%) in the control group reported with history of patching before presented to our clinic. Other information such as age and visual acuity at diagnosis of amblyopia or duration of spectacles was inaccurate and unreliable, so no further investigation and analysis were carried out. 

When conducting research in regards to vision therapy, it is hard to standardize all the naming, instruments and protocols. The first difficulty is the lack of precise criteria for placing a particular instrument in either category [13]. Second, our subjects are children who are become tired and bored more frequently than adults. The scheduled training course might be interrupted unexpectedly. Moreover, vision therapy is similar to other types of therapy that involve learning and education. Motivation and interest influence the effect. Therefore, there are general principles mentioned in treatment program to maintain flexibility instead of hard and fast rules. Single instruments may easy to verify its effect, but multiple and new instruments are more realistic in real world clinical practice. In addition, propose of our study is to demonstrate it. In addition, the vision therapy program is not covered by health insurance in Taiwan. The family chose either vision therapy program or conventional treatment under the consideration of their budget. As a result, we could not assign subjects to case or control group randomly.

Most of the limitations of this study are due to its retrospective nature. It involves few subjects due to the rarity of this subset of the condition. In addition, the lack of completed stereoacuity data makes us hard to draw further conclusion. Compliance with spectacle-wearing and amblyopia training exercises at home could also not be evaluated. However, the effect is valid because compliance with the training program is verified. The diversity of the training programs and the instruments mean that it is difficult to determine which category and which instruments are more effective. More specifically, our vision therapies program which combined orthoptic, perceptual learning, and dichoptic training, so it is unclear which of them are more effective. However, the overall effect of program is positive. A large number of children and a simple training program for a prospective study is required to establish statistically significant results.

## 5. Conclusions

Our vision therapy, which composed of orthoptic therapy, perceptual learning and dichoptic training, is a successful program for improving visual acuity and stereoacuity in 7- to 10-year-old children with bilateral amblyopia that is unresponsive to conventional treatment. 

## Figures and Tables

**Table 1:** Demographic data of case and control group.

	Study (*n*= 15)	Control (*n* = 16)	*p* Value
Age	7.60 (0.74)	7.63 (0.72)	0.924
Sex			0.108
Male	6 (40.0%)	11 (68.8%)	
Female	9 (60.0%)	5 (31.3%)	
History of patching			0.693
	2 (13.3%)	3 (18.8%)	
Anisometropia or Isometropia			1.000
A	4 (26.7%)	5 (31.3%)	
I	11 (73.3%)	11 (68.8%)	
Refractive error			
0 D to +1.00 D	0 (0%)	3 (18.8%)	
+1.00 D to <+2.00 D	0 (0%)	1 (6.2%)	
+2.00 D to <+3.00 D	0 (0%)	1 (6.2%)	
+3.00 D to <+4.00 D	0 (0%)	1 (6.2%)	
+4.00 D to <+5.00 D	0 (0%)	0 (0%)	
≥+5.00 D	1 (6.7%)	2 (12.5%)	
−1.00 D to 0 D	1 (6.7%)	1 (6.2%)	
−2.00 D to <−1.00 D	2 (13.3%)	2 (12.5%)	
−3.00 D to <−2.00 D	0 (0%)	1 (6.2%)	
−4.00 D to <−3.00 D	1 (6.7%)	1 (6.2%)	
−5.00 D to <−4.00 D	0 (0%)	2 (12.5%)	
<−5.00 D	10 (66.7%)	1 (6.2%)	
Depth of Amblyopia			0.3342
severe (>0.7 logMAR)	1 (6.7%)	3 (18.7%)	
moderate (0.3 to 0.7 logMAR)	8 (53.3%)	10 (62.5%)	
mild (<0.3 logMAR)	6 (40%)	3 (18.7%)	
Mean follow up period (m)			0.95
	6.4	6.32	

Values are presented as n (%) or mean (SD).; *p* value for chi square test or two independent *t* test.

**Table 2:** Difference between follow-up and baseline among 2 groups.

Variables	Δ^0−1^			Δ^0−2^			Δ^0−3^			Δ^0−4^			Δ^0-E^		
	C	T	*p*	C	T	*p*	C	T	*p*	C	T	*p*	C	T	*p*
Number	24	30		16	12		8	8		8	4		32	30	
logMAR	0.00 (0.00, 0.08)	0.20 (0.20, 0.33)	<0.001 *	0.00 (0.00, 0.10)	0.20 (0.20, 0.30)	<0.001 *	0.00 (0.00, 0.10)	0.35 (0.20, 0.58)	0.001 *	0.00 (−0.08, 0.00)	0.45 (0.33, 0.58)	<0.001 *	0.00 (0.00, 0.10)	0.30 (0.20, 0.40)	<0.001 *

C: Control group; T: Treatment group; Data presented as median (Q1, Q3). *p* value for Mann-Whitney U test. Δ0-1: Difference between 3rd month and baseline; Δ0-2: Difference between 6th month and baseline; Δ0-3: Difference between 9th month and baseline; Δ0-4: Difference between 12th month and baseline; Δ0-E: Difference between endpoint and baseline; *: *p* value less than 0.05.

**Table 3:** Difference between follow-up and baseline.

	Baseline	3rd m	*p*	Baseline	6th m	*p*	Baseline	9th m	*p*	Baseline	12th m	*p*	Baseline	Endpoint	*p*
Control	0.50 (0.33, 1.00)	0.40 (0.33, 1.00)	0.299	0.40 (0.30, 0.93)	0.50 (0.20, 1.00)	0.777	0.70 (0.30, 1.00)	0.60 (0.20, 1.00)	0.102	0.85 (0.40, 1.00)	1.00 (0.28, 1.00)	0.593	0.50 (0.33, 0.70)	0.40 (0.30, 1.00)	0.613
Case	0.30 (0.20, 0.40)	0.00 (0.00, 0.20)	<0.001 *	0.30 (0.20, 0.40)	0.00 (0.00, 0.18)	0.002 *	0.45 (0.23, 0.65)	0.05 (0.00, 0.10)	0.011 *	0.45 (0.33, 0.65)	0.00 (0.00, 0.08)	0.068	0.30 (0.20, 0.40)	0.00 (0.00, 0.00)	<0.001 *

Data presented as median (Q1, Q3). *p* value for Wilcoxon signed rank test. * Represent *p* value less than 0.05.

## Data Availability

Restrictions apply to the availability of these data. The data are not publicly available due to patient privacy.
